# A clinical study of minimal blood flow clamping combined with 3D printing technology in laparoscopic nephron-sparing surgery

**DOI:** 10.3389/fonc.2024.1520140

**Published:** 2025-01-24

**Authors:** Lingbo Yang, Pengtao Wei, Xiaohui Li, Jiantao Sun

**Affiliations:** Department of Urology Surgery, Luoyang Central Hospital Affiliated to Zhengzhou University, Luoyang, China

**Keywords:** minimal blood flow clamping technology, highly selective renal artery clamping technology, nephron-sparing surgery, renal function, 3D printing technology

## Abstract

**Objective:**

To compare the safety and effectiveness of minimum flow clamping combined with 3D printing technology and highly selective renal artery clamping in laparoscopic nephron-sparing surgery.

**Methods:**

Fifty-two patients with renal cancer undergoing partial resection admitted to Luoyang Central Hospital Affiliated with Zhengzhou University from 2018-01 to 2021-12 were randomly divided into two groups. Group A comprised 30 cases that underwent laparoscopic partial nephrectomy by minimal blood flow clamping combined with 3D printing technology. Group B comprised 22 cases that underwent laparoscopic partial nephrectomy by highly selective renal artery clamping technology. The operation time, renal artery branch clamping time, intraoperative blood loss, postoperative renal function injury, and complication rate were compared between the two groups.

**Results:**

The operation time, blood loss, renal function injury, and complication rate of Group A were shorter than those of Group B (p < 0.05). The time of renal artery clamping in Group B was shorter than that in Group A (p < 0.05). There was no statistical significance in the positive rate of resection margin between the two groups (p > 0.05).

**Conclusions:**

In laparoscopic nephron-sparing surgery, minimum blood flow clamping technology not only can meet the requirements of accurate tumor resection but also can maximize the preservation of residual renal function, greatly shorten the operation time, reduce the amount of blood loss, and reduce the incidence of perioperative complications, making it worthy of promotion in clinical work. Clinical Trial Registration: [website], identifier [registration number].

## 1 Introduction

Renal cell carcinoma is a common malignant tumor in the urinary system, with more men than women affected, and the age of onset is often between 50 and 70 years. The proportion of small renal cell carcinoma has been increasing year by year (1). With the development of laparoscopic technology, domestic and foreign guidelines recommend that T1 tumors should be treated with precision under the premise of complete tumor resection, and the nephron-sparing surgery should be prioritized (2). Compared with the traditional laparoscopic nephrectomy, the selective renal artery clamping and minimal blood flow clamping technology in partial nephrectomy have obvious advantages: can maximize the preservation of residual nephron tissue and minimize the nephron injury related to ischemia. 3D printing technology is a method of building three-dimensional solid structures of objects using software to establish a physical model based on CT data and then printing the objects layer by layer using plastic or fine metal powder, etc. Surgeons use the three-dimensional structure to clearly identify the location of the tumor, blood supply, and resection margin (3). This study selected the data of T1–T2 stage renal cell carcinoma patients from our hospital and used two different technologies combined with 3D printing to perform laparoscopic nephron-sparing surgery. The safety and effectiveness of the two different surgical methods were compared, and the specific report is as follows.

## 2 Materials and methods

### 2.1 Case data

A total of 52 kidney cancer patients who underwent surgery at Luoyang Central Hospital from January 2018 to December 2021 were selected and divided into two groups: before surgery, 3D printing technology was used to precisely locate the shape, location, and blood circulation of the affected kidney and tumor. The cost of a 3D printed model is approximately 400 yuan, which can also be included in medical insurance, divided into two groups: Group A comprised 30 cases that underwent laparoscopic nephron-sparing surgery using minimal blood flow clamping technology., and Group B comprised 22 cases that underwent laparoscopic nephron-sparing surgery performed using highly selective renal artery clamping technology. Inclusion criteria were as follows: all patients undergo preoperative examination without distant metastasis or lymph node metastasis and without venous cancer thrombus. Preoperative liver and kidney function-related indicators were normal. All enrolled patients have no history of valve replacement or are taking antiplatelet or anticoagulant drugs. All patients were given R.E.N.A.L score. All 52 patients underwent surgery by the same experienced surgeon. Patients with preoperative liver and kidney dysfunction, moderate or above anemia, difficult-to-control diabetes, hypertension, and heart failure were excluded. The pathology of all enrolled patients was clear cell carcinoma. There was no statistically significant difference (p > 0.05) in the basic clinical data between the two groups of patients, as shown in **Table 1**.

**Table 1:** Comparison of baseline characteristics.

Group	Gender		n	BMI (kg·m^−2^)	Year	RENAL		Underlying diseases	Preoperative serum hemoglobin (g/L)	Tumor size (cm)
	M	F								
Group A Group B	14 9	16 13	30 22	24.99 (21.47, 25.80) 25.16 (22.31, 26.67)	59 (54, 76) 62 (53, 74)	5.20 ± 1.32a 6.14 ± 2.82a	4.96 ± 1.22p 4.84 ± 1.62p	22 17	116 ± 19.44 122 ± 21.08	3.64 (2.5, 5.4) 3.7 (3.0, 5.2)
T/x^2^ p	>0.05			>0.05	>0.05	>0.05		>0.05	>0.05	>0.05

BMI, body mass index.

### 2.2 Surgical methods

**Figure 1:**
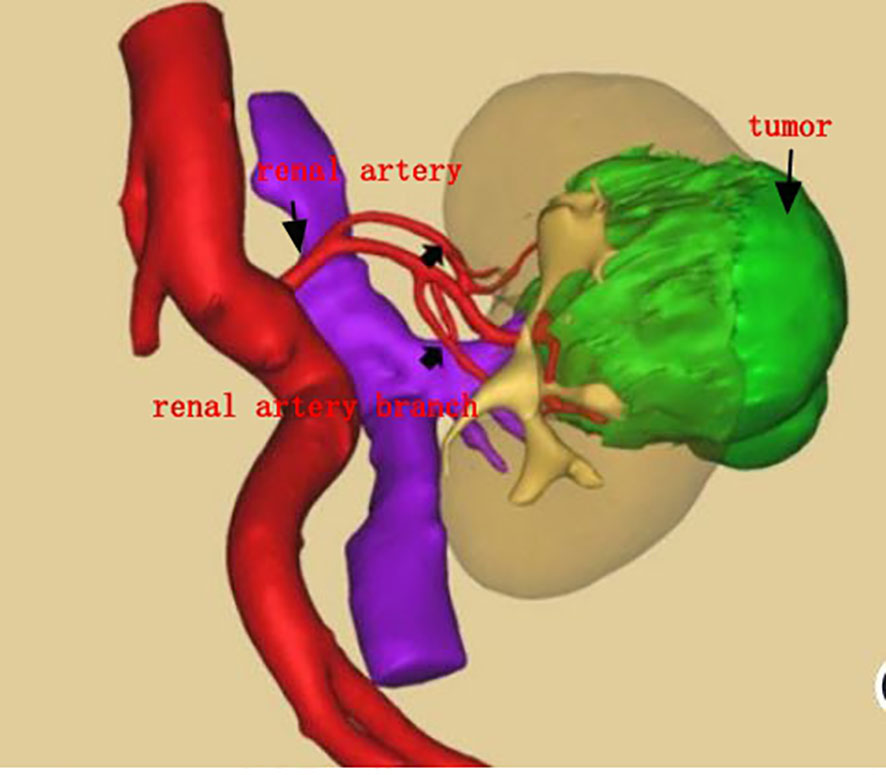
Three-dimensional (3D) model of kidney tumor.

**Figure 2:**
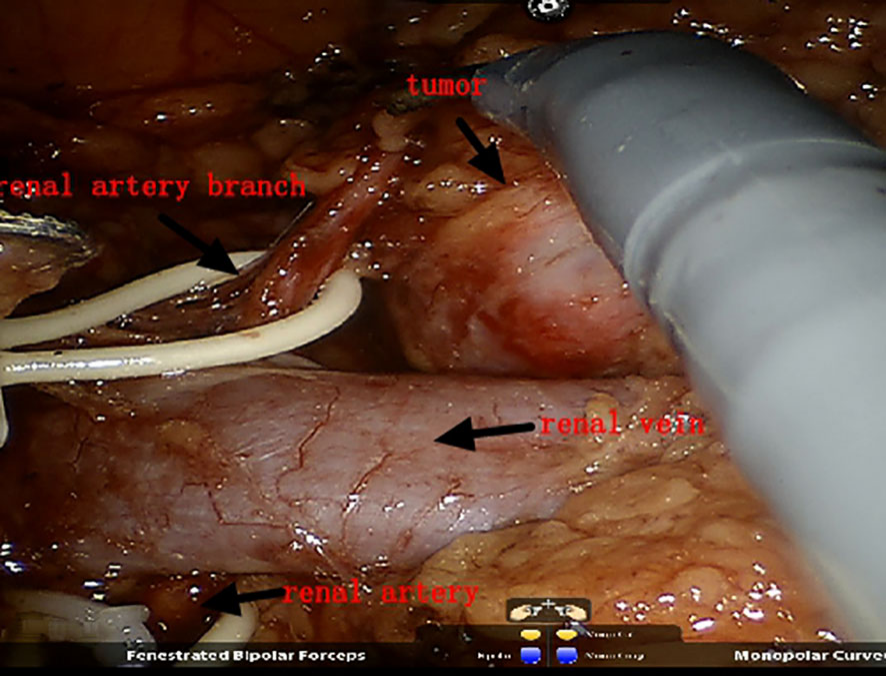
Exposing the main renal artery and branch arteries.

**Figure 3:**
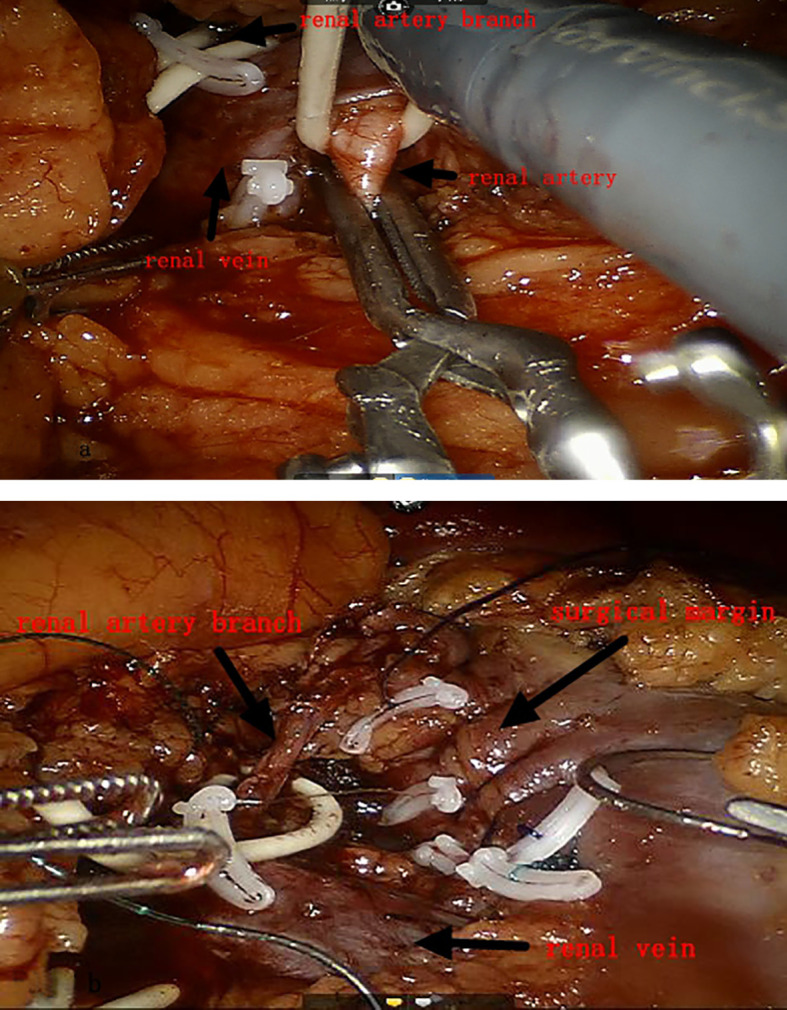
**(A)** Main renal artery and branch arteries are clamped at the same time. **(B)** Opening the main renal artery and branch arteries in steps.

**Figure 4:**
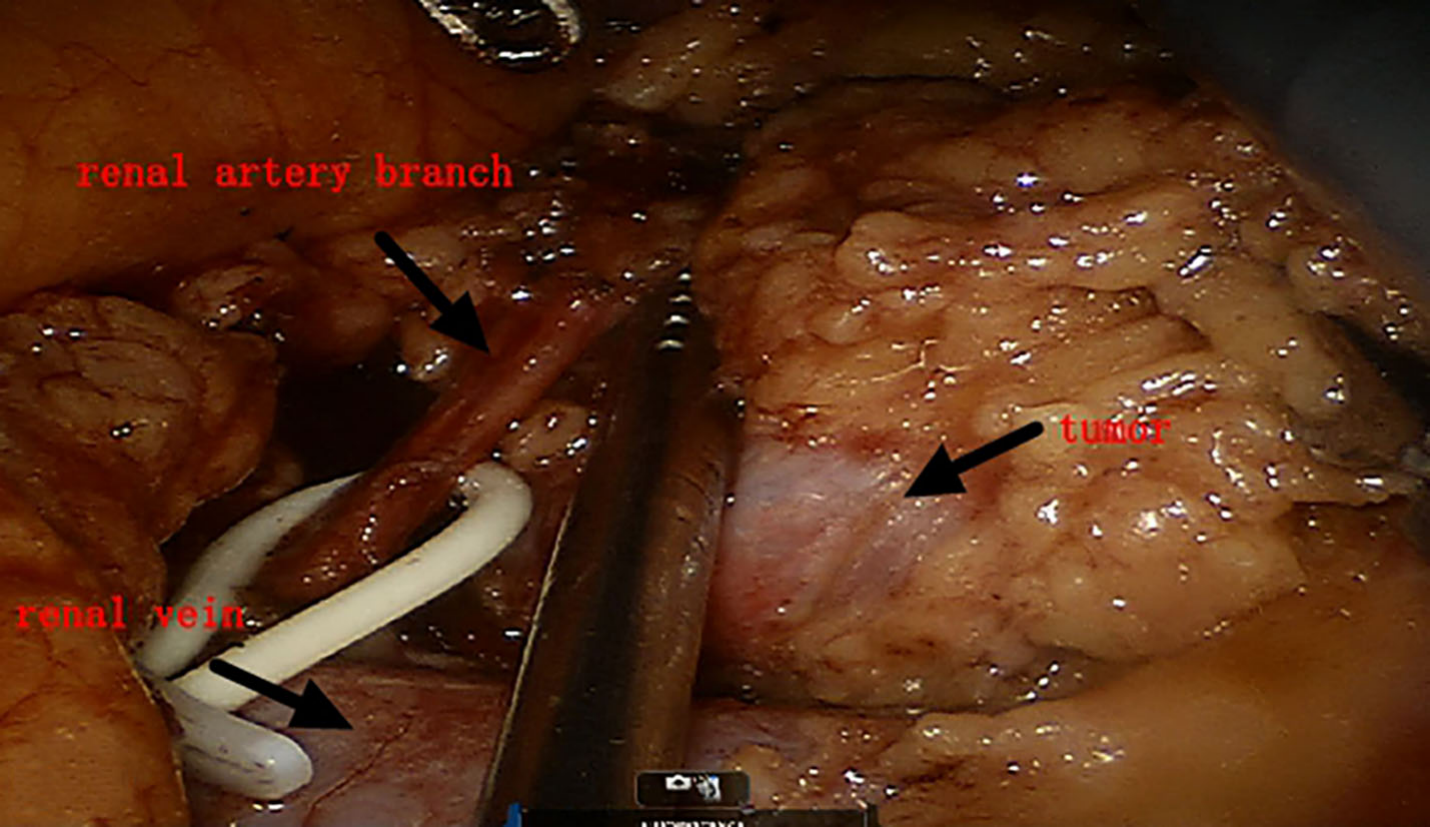
Blocking only the branch artery of the renal artery.

Both groups of patients underwent surgery via the intraperitoneal route: the preparation work was the same in the early stage. The patients were placed under general anesthesia in a lateral position, and after successfully establishing pneumoperitoneum, instruments were used. The lateral peritoneum was incised in the spleen area (left surgery) or the liver area adjacent to the colon (right surgery), and the intestinal tract was pushed inward to fully expose the kidney. First, the renal blood vessels were revealed, and then the surrounding tissue of the tumor was freed to expose the tumor, fully exposing the main renal artery and the branch arteries nourishing the tumor area (**Figures 1**, **2**). In Group A, it was ensured that the main and branch arteries of the renal artery were clamped at the same time before the tumor resection, and zero bleeding was achieved during complete tumor resection. After the inner cutting edge was sutured using a V-lock barbed shape suture, the renal artery was opened as soon as possible, the branch artery vessel was continuously clamped, and then the branch artery was opened after suturing on the outer side of the margin. If there was bleeding from the margin, suture 1 or 2 needle or spray biological protein glue was intermittently added (**Figures 3A, B**). In Group B, before tumor resection, it was ensured that the renal artery branch was clamped, and then time started. Tissue scissors were used to completely remove the tumor along the circumference of 0.5–1.0 cm (**Figure 4**). After the V-lock barbed shape suture was used to suture the surgical margin, the arterial clamp clip was released. If there was bleeding from the wound, biological protein glue was sprayed, and a drainage tube was left in the abdominal cavity after surgery to close the incision.

### 2.3 Observation indicators

The following were the observation indicators: surgical time, bleeding volume, positive margin rate, incidence of complications [postoperative fever (body temperature >38°C), bleeding volume >400 mL, nephrectomy, urinary leakage, renal dysfunction, wound infection, cardiovascular and cerebrovascular accidents, etc.], renal artery clamping time, renal function injury, postoperative gastrointestinal function recovery time, and pain score on the first day after surgery.

### 2.4 Statistical methods

The measurement data that did not conform to a normal distribution were expressed as the median (lower quartile, upper quartile), and the non-parametric test was used for the comparison between groups. The measurement data that conformed to a normal distribution were described by mean ± standard deviation (SD), and the t-test was used for the inter-group comparison. The statistical data were expressed as rates (%), and the X^2^ test was used for the comparison between groups. p < 0.05 indicates a statistically significant difference. All the statistical analyses were conducted using SPSS 26.0 (IBM Corporation, Armonk, NY, USA).

## 3 Result

All surgeries in Group A were successfully completed, while one patient in Group B underwent radical nephrectomy due to massive ongoing blood loss during the operation. One patient had a positive surgical margin. Group A had advantages over Group B in terms of surgical time, intraoperative blood loss, postoperative complications [postoperative fever (body temperature >38°C), blood loss >400 mL, nephrectomy, urinary leakage, renal dysfunction, wound infection, cardiovascular and cerebrovascular accidents, etc.], short-term renal function injury, and postoperative first-day pain score with statistical significance (p < 0.05). Group B had an advantage over Group A in terms of renal artery clamping time, and the difference was statistically significant (p < 0.05). There was no statistically significant difference in the positive rate of surgical margins and postoperative expelling flatus time between the two groups (p > 0.05). A total of 52 patients in this group were followed up routinely for 24–48 months, and no tumor recurrence or metastasis was observed. One month after surgery, the renal function of the two groups of patients returned to normal, which was mainly verified by rechecking blood creatinine. Postoperative rechecking of blood biochemistry was simple and cost-effective, while glomerular filtration rate (GFR) testing was too expensive to be clinically promoted, as shown in **Tables 2**, **3**.

**Table 2:** Comparison of measurement data.

	Operation time (min)	Operative bleeding volume	Complications	Increased creatinine	Blocking time (min)	Incisal positive n%	Expelling flatus time	Postoperative pain score after 1st day
Group A	55.36 ± 14.62	30.17 (21.47, 55.80)	3.14 ± 2.04	36.02 ± 22.66	20.11 ± 6.38	0	1.24 ± 1.04	4.11 (2.047, 55.80)
Group B	68.22 ± 11.33	55.69 (30.55, 95.80)	6.21 ± 3.11	54.02 ± 20.66	18.28 ± 3.22	0	1.54 ± 1.74	5.11 (2.966, 7.108)
T/x^2^ p	0.42 0.02	0.02	0.519 0.01	0.28 0.01	0.48 0.03		0.11 0.07	0.04

**Table 3:** The complications with a Clavien Dindo classification.

Group classification	I, n%	II, n%	III, n%		IV, n%		V, n%	Summation
			IIIa	IIIb	IVa	IVb		
Group A	20 (66.7)	4 (13.3)	0	0	0	0	0	24 (80)
Group B	12 (54.5)	5 (22.7)	2 (9.1)	1 (4.5)	1 (4.5)	0	0	21 (95.5)
p	>0.05	0.004						0.023

## 4 Discussion

Renal cell carcinoma is a common malignant tumor in the urinary system. With the development of technology, the probability of screening renal cell carcinoma is increasing, especially with a higher proportion of T1-stage tumors. At present, precision treatment for tumors nephron-sparing surgery has become a standard surgical method recommended by major guidelines at home and abroad, and its indications have further expanded with the development of minimally invasive technology (4). Wang Yongliang and his team proposed that laparoscopic nephron-sparing surgery may be considered for T2-stage tumors (5). Professor Cui Xingang clearly stated that laparoscopic nephron-sparing surgery is also beneficial for kidney cancer patients with special tumor locations, which is consistent with the results of the study. Multiple medical centers have confirmed that localized renal tumors and early nephron-sparing surgery have no statistically significant difference in long-term survival rate compared to radical nephrectomy. However, early laparoscopic nephron-sparing surgery can maximize the preservation of functional nephrons, minimize the loss of renal function, and greatly reduce the incidence of adverse renal events (6). In the study, we found that the use of minimal blood flow obstruction technology in laparoscopic nephron-sparing surgery can further shorten the surgical time, reduce intraoperative bleeding, and lower the incidence of perioperative complications while ensuring accurate and complete tumor resection.

3D printing technology is widely used in medicine, including designing surgical paths and defining surgical resection ranges. Compared to conventional CT, 3D printing technology can more intuitively reveal the specific location of the renal tumor, as well as the relationship between the tumor supply branch arteries and adjacent organs such as the ureter. These advantages help surgeons to more accurately locate the renal tumor during surgery, quickly clamp the branch artery, accurately remove the depth, and preserve the renal function to the greatest extent possible (7). With the help of 3D printing models, surgeries can be completed more accurately, resulting in shorter hospital stays, fewer complications, and a decrease in total perioperative costs.

We believe that the key to using 3D printing combined with minimal blood flow obstruction technology for laparoscopic nephron-sparing surgery is to first ensure that the main and branch arteries of the renal are clamped together before tumor resection so as to achieve real “zero bleeding” during the complete resection of the tumor. This not only ensures the principle of complete tumor resection but also shortens the tumor resection time. After suturing the inner cutting edge with a V-lock barbed suture, the main renal artery must be opened early, which can ensure that the warm ischemia of residual nephron is short and the branch artery is continuously clamped. After suturing the outside of the surgical margin, the branch artery will be opened. The clamped renal artery and renal artery branch must be opened in an orderly manner so as to achieve maximum preservation of residual renal tissues and the shortest time of warm ischemia. How can we preserve as many nephrons as possible in practical clinical work? Some scholars believe that the tumor should be removed at a distance of 10 mm from the tumor margin, while others suggest that a distance of 1–2 mm from the tumor margin is optimal (8). Currently, the specific value of the distance from the tumor margin is not yet determined, but we believe that the distance of 5 mm from the tumor margin is optimal. This can ensure negative margins, achieve the principle of preserving nephrons without tumors, and preserve as many normal nephrons as possible. Of course, it is important to carefully search for any large blood vessels and any damage to the collecting system after tumor excision. If there is any damage, immediate repair is necessary. If the surgical margin is deep after tumor resection, it is recommended to suture it in two layers. It is important to note that the inner layer should not be sutured too deeply and that the suture should not be pulled too hard to avoid laceration of the renal parenchyma. At the same time, the needle should be inserted from one side of the blood vessel during suturing, and more attention should be paid to avoid accidental damage to the renal portal system. For some renal portal tumors, there are fewer residual normal renal tissues in the renal portal area after tumor excision. It is recommended to suture the surgical margin by closing to the tumor bed after tumor excision and use continuous suturing of the renal parenchyma incision. The renal capsule must be included during the running suture, which can maximize the anatomical reconstruction of the surgical incision and minimize the laceration of renal parenchyma due to postoperative arterial congestion. At the same time, it can significantly reduce the occurrence of anastomotic leakage. Of course, a small number of patients may experience urinary leakage after surgery. In such cases, it is generally recommended to place a ureteral stent. Domestic and foreign literature mostly recommends a placement time of 4–12 weeks, and a very small number of patients still experience urinary leakage after 12 weeks. It is recommended to undergo excision of the fistulous tract following anastomosis and kidney repair surgery. Continuous hematuria caused by anastomosis bleeding or laceration of the collecting system during the perioperative period is also the most concerning situation for urologists. Its occurrence is mainly related to the extent of tumor invasion, surgical resection depth, laceration of the renal pelvis system, and anatomical reconstruction of the surgical incision after surgery. Therefore, for endogenous tumors and tumors closing to the renal portal area, we can first consider using minimal blood flow clamping technology to perform nephron-sparing surgery. This not only allows for precise tumor resection but also reduces the risk of delayed surgery due to poor visibility and improves the chances of reducing bleeding.

However, there are a small number of cases having larger tumors resulting in significant loss of renal tissue after tumor excision. Internal suturing after the tumor excision may not be able to anatomically align the tissue, which leads to persistent hematuria, and even blood clots and bladder clot tamponade. Therefore, if the condition permits, emergency treatments such as cystoscopic blood clot removal, continuous bladder irrigation, and the use of hemostatic agents or absorbable hemostatic materials to fill the cavity (9) should be administered as soon as possible. It is advisable to restore the anatomical position of the kidney and suture the renal perinephric fascia again, which can exert a certain degree of compression on the resection margin and reduce the incidence of bleeding to some extent. Of course, we can also use laparoscopic ultrasound for precise tumor localization (10), which can provide more precise targeting of the tumor. In this study, all two patients with renal pelvis tumors were treated with minimal blood flow occlusion techniques, accompanied by preoperative 3D printing technology, and the tumors were precisely resected. All resection margins were negative after surgery, and no recurrence was observed during 24-month follow-up, which is consistent with the views of Di Pierro and his team (11).

It is generally believed that the key to nephron-sparing surgery is the control of renal blood vessels (12). Patients with renal warm ischemia time >25 minutes will experience irreversible damage to renal function after surgery, while patients with warm ischemia time <25 minutes will have little long-term impact on renal function (13). This is consistent with the viewpoint of Chu Jian and his team (14). Meanwhile, some scholars have also found that for patients undergoing preservation of renal unit surgery, the risk of acute renal failure after surgery increases by approximately 5% for each additional minute of renal warm ischemia time (15). Therefore, it is advisable to minimize renal warm ischemia time under the premise of ensuring safety and tumor-free principle. Additionally, the amount of kidney tissue preserved has a more significant impact on the patient’s postoperative renal function. This view is fully consistent with that of Liu Pengfei and others (16). In this study, 30 patients underwent nephron-sparing surgery using minimal blood flow clamping technology. Although the renal artery was blocked for a longer time than the choice of renal artery branch blockage, the former could ensure a “zero bleeding” operation, guarantee a clear surgical field, accurately remove the tumor, and maximize the preservation of normal kidney tissue volume under the premise of tumor-free principle.

Therefore, the postoperative 1-week renal function of the group with minimal renal artery blood flow clamping was higher than that of the group with highly selective renal artery clamping, with statistical significance, which may be due to the fact that more renal units were preserved. Postoperative 1-month, 3-month, and 6-month renal function checks showed no significant difference in the long-term effects of the two surgical methods. This also shows that the reserve function of the kidney allows for the recovery of renal function after surgery, and the short-term renal function can remain relatively stable. This is consistent with the research results of the Solomon LW team (17). Additionally, there was a statistically significant difference in the incidence of complications between the groups (p = 0.023), with this difference being more pronounced at grade II and above, which proves the advantage of minimal blood flow obstruction technology in preserving nephrons once again. The results of this study are inconsistent with Professor Riccardo Schiavina’s opinion (18); the main reason is that he did not compare different blood flow obstruction methods for nephron-sparing surgery, while this study explored the prognosis of two different surgical methods, so there is a certain difference in the results.

In summary, the use of minimal renal artery blood flow clamping combined with 3D printing technology for laparoscopic nephron-sparing surgery is safe and reliable. Especially for complex tumors, large tumors, or tumors closing to the renal portal area, it has certain advantages. The results of this study are inconsistent with Professor Mario Belmonte’s viewpoint (19), mainly due to the different blocking methods involved in this study and the different correlation between blocking and opening blood flow time. Compared with traditional surgery, it can significantly shorten surgical time, reduce bleeding, and lower the incidence of complications and positive margins, making it worthy of further promotion in clinical practice.

## Data Availability

The original contributions presented in the study are included in the article/**Supplementary Material**. Further inquiries can be directed to the corresponding author.
